# Telomere maintenance-related genes are important for survival prediction and subtype identification in bladder cancer

**DOI:** 10.3389/fgene.2022.1087246

**Published:** 2023-01-06

**Authors:** Yonggui Xiao, Danping Xu, Chonghao Jiang, Youlong Huili, Shiwen Nie, Hongfei Zhu, Guorui Fan, Xiaohai Guan

**Affiliations:** ^1^ Affiliated Hospital of North China University of Science and Technology, Tangshan, China; ^2^ Sichuan Academy of Medical Sciences and Sichuan Provincial People’s Hospital, Chengdu, China

**Keywords:** bladder cancer, telomere maintenance-related genes, prognostic models, subtypes, nomogram

## Abstract

**Background:** Bladder cancer ranks among the top three in the urology field for both morbidity and mortality. Telomere maintenance-related genes are closely related to the development and progression of bladder cancer, and approximately 60%–80% of mutated telomere maintenance genes can usually be found in patients with bladder cancer.

**Methods:** Telomere maintenance-related gene expression profiles were obtained through limma R packages. Of the 359 differential genes screened, 17 prognostically relevant ones were obtained by univariate independent prognostic analysis, and then analysed by LASSO regression. The best result was selected to output the model formula, and 11 model-related genes were obtained. The TCGA cohort was used as the internal group and the GEO dataset as the external group, to externally validate the model. Then, the HPA database was used to query the immunohistochemistry of the 11 model genes. Integrating model scoring with clinical information, we drew a nomogram. Concomitantly, we conducted an in-depth analysis of the immune profile and drug sensitivity of the bladder cancer. Referring to the matrix heatmap, delta area plot, consistency cumulative distribution function plot, and tracking plot, we further divided the sample into two subtypes and delved into both.

**Results:** Using bioinformatics, we obtained a prognostic model of telomere maintenance-related genes. Through verification with the internal and the external groups, we believe that the model can steadily predict the survival of patients with bladder cancer. Through the HPA database, we found that three genes, namely ABCC9, AHNAK, and DIP2C, had low expression in patients with tumours, and eight other genes—PLOD1, SLC3A2, RUNX2, RAD9A, CHMP4C, DARS2, CLIC3, and POU5F1—were highly expressed in patients with tumours. The model had accurate predictive power for populations with different clinicopathological features. Through the nomogram, we could easily assess the survival rate of patients. Clinicians can formulate targeted diagnosis and treatment plans for patients based on the prediction results of patient survival, immunoassays, and drug susceptibility analysis. Different subtypes help to further subdivide patients for better treatment purposes.

**Conclusion:** According to the results obtained by the nomogram in this study, combined with the results of patient immune-analysis and drug susceptibility analysis, clinicians can formulate diagnosis and personalized treatment plans for patients. Different subtypes can be used to further subdivide the patient for a more precise treatment plan.

## 1 Introduction

According to statistics, the incidence of bladder cancer is increasing year by year, and its incidence ranks among the top ten among all tumours. Because of smoking, hormones, and other factors, the incidence of bladder cancer is higher in men, ranking sixth among all tumours in men (Kamat et al., 2016; Lenis et al., 2020; Siegel et al., 2021; Sung et al., 2021). Patients with bladder cancer have poor quality of life and high mortality, with a 5-year overall survival rate as low as 23% (Antoni et al., 2017; Lee et al., 2018; Kuroki and Guntupalli, 2020). Advanced age, hair colouring, and smoking are all associated with bladder cancer, with smoking increasing the risk of bladder cancer up to six-fold (Shariat et al., 2010; Freedman et al., 2011; McKiernan and Asafu-Adjei, 2017). At present, the conventional treatment method is surgical resection with adjuvant chemotherapy and immunotherapy, but the postoperative recurrence rate of bladder cancer in patients is high, and the effect of adjuvant chemotherapy and immunotherapy is limited, so it is extremely important to explore new treatment methods (Kamat et al., 2016). Due to the complex causes of bladder cancer, and its high degree of heterogeneity, the traditional staging system has limited clinical role (Gerlinger et al., 2015; Kluth et al., 2015). Therefore, there is an urgent need to develop a new prognostic model for predicting the survival of patients with bladder cancer, so that clinicians can develop different treatment plans for patients with different survival periods.

Telomeres are specific nucleoprotein structures composed of TTAGGG nucleotide repeats. These special structures protect the ends of chromosomes, and are often referred to as the biological clock of cell division (Piqueret-Stephan et al., 2016). Studies have found that most tumour cells achieve immortality by counteracting telomere shortening, through the telomere maintenance mechanism (TMM) (Shay and Wright, 2001). In addition, telomeres are also associated with heart disease, congenital dyskeratosis, diabetes, and other diseases (Elks and Scott, 2014; Maciejowski and de Lange, 2017; Savage, 2018). Relevant studies have found that 60%–80% of patients with bladder cancer are affected by telomerase reverse transcriptase promoter (TERT) mutations (Bell et al., 2016; Günes et al., 2018; Lopez-Beltran et al., 2021). As early as 2018, a review by Ricardo Leão et al. described in detail the various genetic and epigenetic mechanisms that lead to the upregulation of hTERT in tumors, and pointed to its strong potential as a biomarker (Leao et al., 2018). In a further study by Ricardo Leão et al., in 2019, it was confirmed that the genetic and epigenetic combination of the TERT promoter was able to predict the prognosis of NMIBC (Leao et al., 2019).

Through previous research, we have found that telomere maintenance genes are closely related to the occurrence and development of tumours, and bladder cancer is no exception. In-depth research on the relationship between telomere maintenance genes and bladder cancer combined with bioinformatics is expected to find new diagnostic indicators, to provide a reference for clinicians to diagnose and formulate treatment plans. In this study, we calculated a prognostic model of telomere maintenance genes for bladder cancer, using bioinformatics techniques. By integrating the model formula scoring with clinical information, we obtained a nomogram, which enabled the prediction of the survival rate of patients with bladder cancer to be easily calculated. As a result, clinicians can formulate diagnosis and personalized treatment plans for patients, based on the results of patient survival prediction, immunoassays, and drug susceptibility analysis.

## 2 Materials and methods

### 2.1 Acquisition of data

We downloaded the raw data for bladder cancer from the TCGA database and collated the data in R (4.2.1) and Perl (strawberry version). The external validation data came from the GSE32894 dataset of the GEO database. The immunohistochemical results of model-related genes were queried in the HPA database. In addition, we obtained telomere maintenance genes through the TelNet website.

### 2.2 Screening and analysis of prognostically relevant differential genes

We obtained 2093 Telomere maintenance-related genes through the TelNet database. Differential genes in tumours and normal tissues were obtained using limma packages (Filter is fdr < 0.05, |logFC| > 1). The differential genes obtained by screening were used for single-factor independent prognostic analysis. We used the sva package to standardize TCGA and GEO data, to remove batch effects, after which TCGA expression data were combined with survival information. Univariate independent prognostic analysis was performed to obtain prognostically relevant differential genes (*p* < 0.005). The copies and mutations of prognostically related genes were analysed.

### 2.3 Model construction and validation

Taking the TCGA cohort as the internal group, the GEO cohort built the lasso regression model for the external group. The model was then cross-validated, and the best result was selected to output the model formula and obtain the model-related genes. Then, all samples in the internal group and the external group were scored by the model formula, to obtain the risk score. All samples were divided into A and B groups, based on the median risk score of the internal group (Group A was high risk, and Group B low risk). Finally, the survival analysis of the internal and external groups was carried out to test the stability of the model. Then, progression-free survival analysis and ROC curve were used to test the accuracy of the model. Univariate and multivariate independent prognostic analysis was used to assess whether the risk score could be used as an independent prognostic indicator. The HPA database was again used to query the immunohistochemistry of model genes.

### 2.4 Validation of the predictive power of the model in different populations

To confirm the generalizability of the model, we first analysed the correlation of the model with clinicopathological information in patients with bladder cancer. Patients were grouped according to clinicopathological information and survival analysis performed, to assess whether the model worked for different populations.

### 2.5 Construction and calibration of nomograms

To render model use more intuitive to clinicians, we drew a nomogram that integrated the age, tumour stage, and risk score of patients with bladder cancer. Next, we evaluated the accuracy of the nomogram, by comparing the predicted and actual values in the calibration curve. Further independent prognosis analysis was carried out on the nomogram, to assess whether the nomogram was not affected by other factors.

### 2.6 Pathway enrichment analysis

Firstly, the enrichment of the pathway was analysed by GSVA. Then, we used KEGG and GO for the enrichment of the pathway.

### 2.7 Immune-related analysis

We first looked at the relationship between immune cell infiltration and risk scores through seven different analytical platforms (TIMER, CIBERSORT, XCELL, QUANTISEQ, MCPCOUNTER, EPIC, and CIBERSORT). The microenvironments of the tumours were analysed by the estimation package. Then, ssGSEA was used to measure the infiltration of immune cells, and the enrichment of immune-related functions in the high- and low-risk groups. Finally, the differences in the expression of immune checkpoint-related genes between high- and low-risk groups were studied.

### 2.8 Drug susceptibility analysis

Using the pRRophetic package, the semi-maximum inhibitory concentration (IC_50_) of the targeted drug was predicted by gene expression levels to reflect therapeutic sensitivity.

### 2.9 Identification and analysis of different subtypes

To explore personalized treatment of bladder cancer, we divided samples into different subtypes through the ConsensusClusterPlus package, based on the expression of the 11 model-related genes. Survival analysis and clinical correlation analysis were then performed for different subtypes. Plotting Sankey to understand the distribution of different subtypes and high- and low-risk groups. In order to explore the immune characteristics of different subtypes, we also performed immune-related analysis on different subtypes. Finally, we performed drug susceptibility analyses for different subtypes.

### 2.10 Statistical analysis

All statistical analyses are performed using perl software and R software (version 4.2.1, packages: sva, limma, pheatmap, survival, survminer, maftools, RCircos, glmnet, timeROC, ggpubr, regplot, rms, ConsensusClusterPlus, estimates, scales, ggplot2, ggtext, reshape2, tidyverse, GSEABase, and pRRophetic). Unless otherwise stated, for all analyses in this study the estimated *p*-value was < 0.05. For all comparisons: “*” *p* < 0.05, “**” *p* < 0.01, “**” *p* < 0.001.

## 3 Results

### 3.1 Seventeen prognostically relevant differential genes were obtained

**FIGURE 1:**
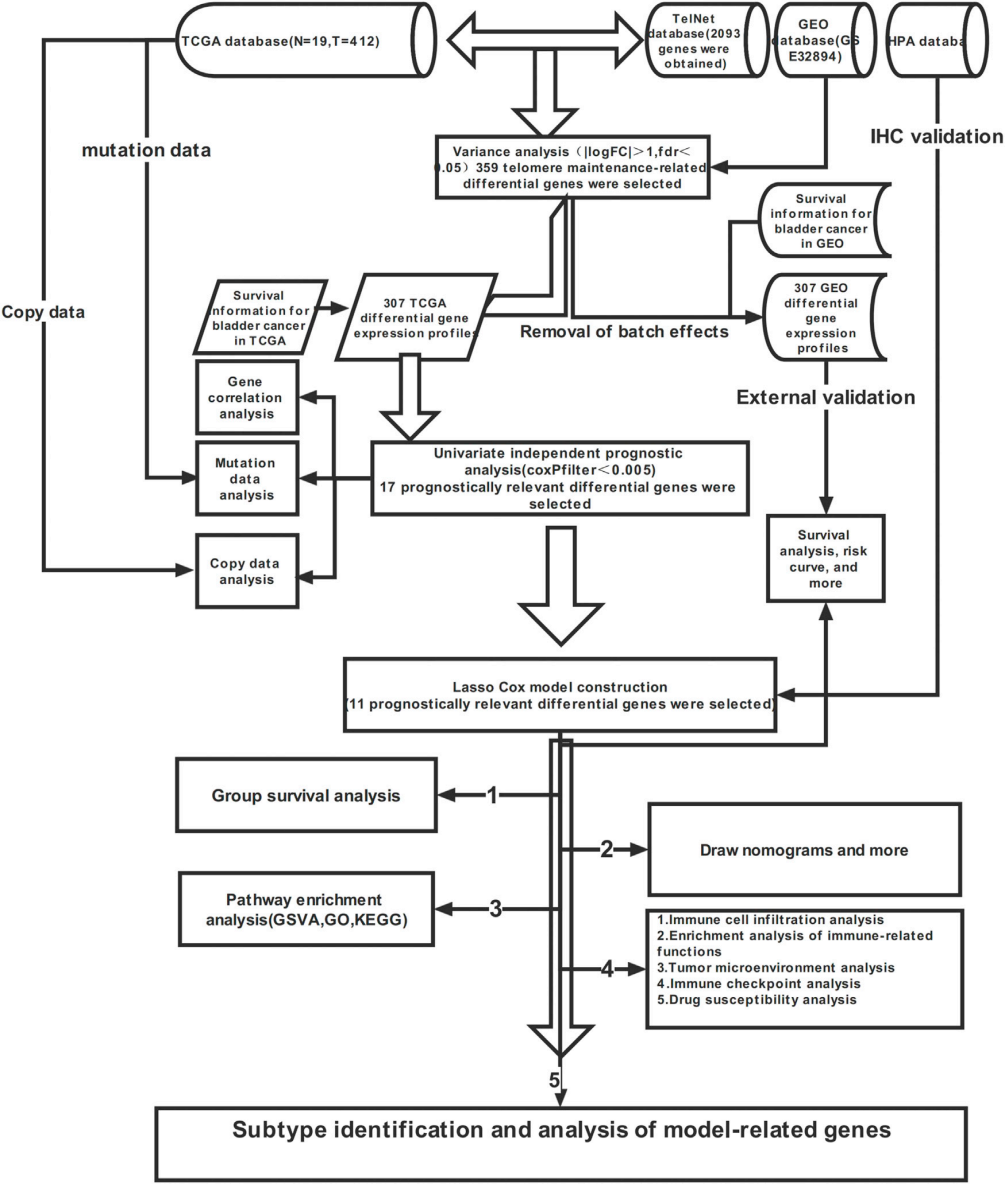
Flow chart of this study.

**FIGURE 2:**
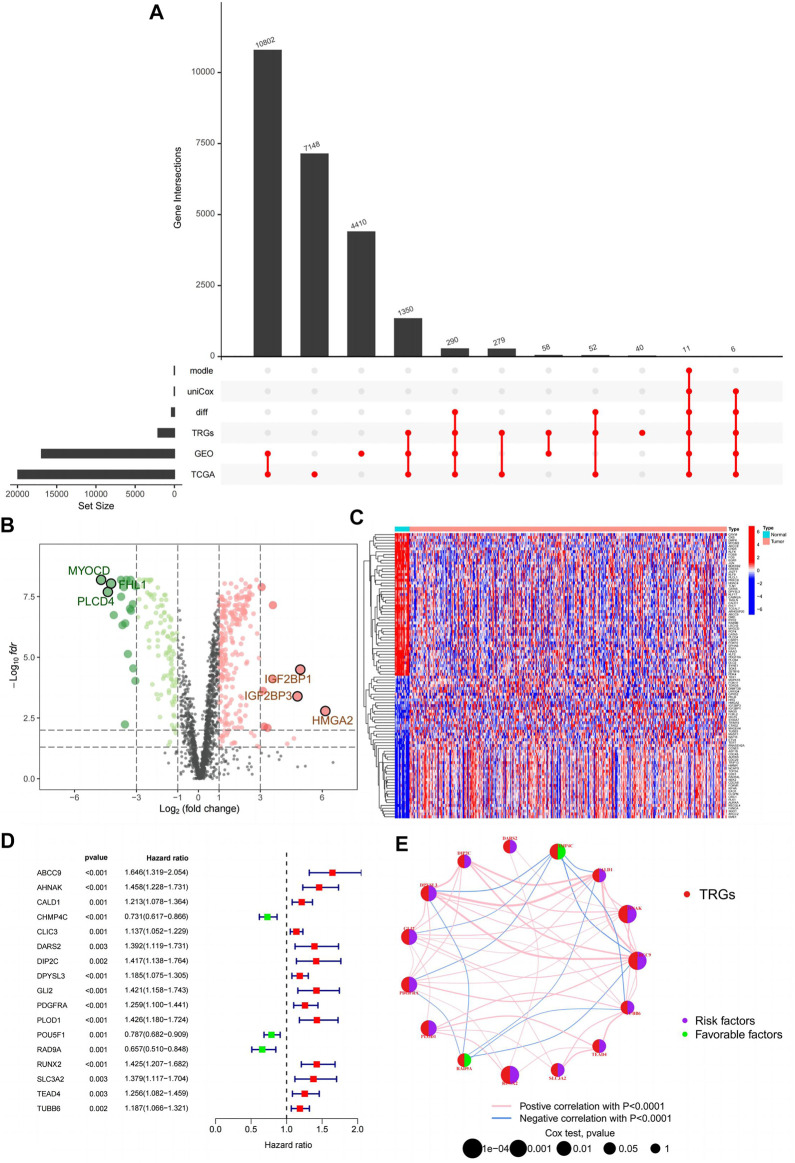
Prognostically relevant differential genes are obtained. **(A)** Upset plot for this study; **(B)** Volcanic map of 359 differentially expressed telomeres maintained relevant genes; **(C)** Heatmap of 359 differentially expressed telomeres maintained relevant genes; **(D)** Forest plot of 17 prognostically relevant telomere maintenance-related genes; **(E)** Prognostic network diagram of 15 prognostically relevant telomeres maintenance-related genes.

First, we demonstrate the entire process of the study (Figure 1; Figure 2A). We obtained 1,725 differentially expressed genes in tumours and normal tissues through differential analysis. In the volcano map, the 1,725 differential genes are at different locations. We obtained 359 significantly different genes (|logFC| > 1, Fdr < 0.05), of which 66% were upregulated in red, 34% were downregulated in green, and the rest were represented in black (Figure 2B). We selected the 50 points farthest from the coordinate axis in the red and green regions of the volcano map, to plot the heat map (Figure 2C). The expression spectrum of the resulting differential genes was standardized with GSE32894, to remove batch effects. The expression profiles of 307 genes were obtained, and then combined with survival information, to obtain 17 prognostically relevant differential genes, of which 14 were HR > 1 and 3 HR < 1 (Figure 2D). Through the prognostic network diagram, we found that 15 genes were co-expressed (Figure 2E, *p* < 0.0001).

### 3.2 Co-expression and mutations and copies of prognostically relevant differential genes

**FIGURE 3:**
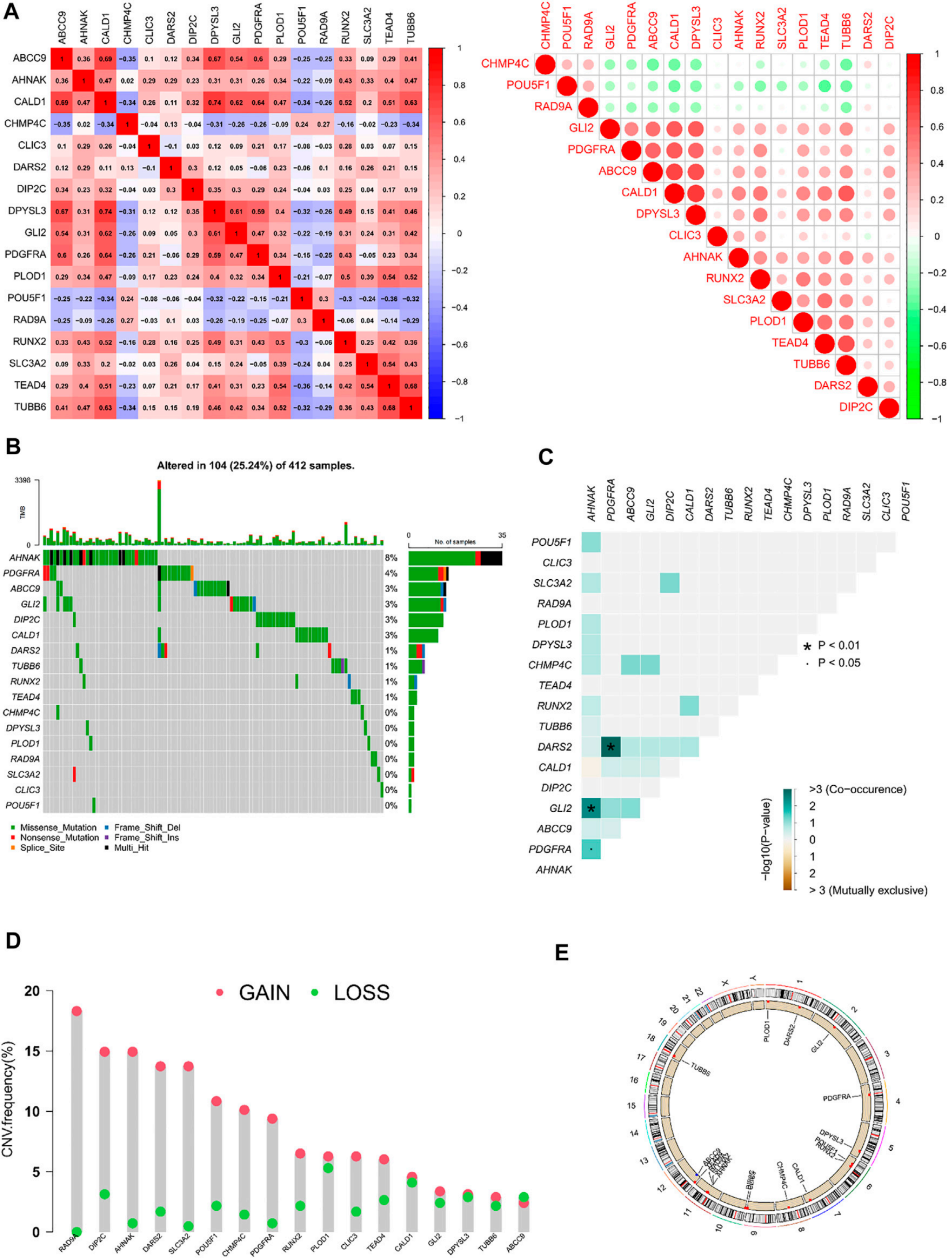
Correlation analysis and mutations and copies of prognostically differential genes **(A)** Heatmap of the correlation of 17 prognostically relevant differential genes; **(B)** Waterfall plot of 17 prognostically relevant differential genes; **(C)** Co-mutation heatmap of 17 prognostically prognostically relevant differential genes; **(D)** Statistical chart of copy frequencies of 17 prognostically related differential genes; **(E)** Copy number circle plot of 17 prognostically relevant differential genes.

The co-expression, mutations, and copies of prognostically related differential genes were further analysed. The relationship between the 17 prognostically relevant genes was mainly positive, with CALD1 and DPYSL3 exhibiting the strongest correlation (Figure 3A). Mutation analysis found that 10 of the 17 genes were mutated, with the AHNAK gene being the most frequently mutated, and the most frequent mutation type being missense (Figure 3B). Interestingly, the AHNAK gene co-mutates with 11 of the other 16 genes, and there are also significant co-mutations between the PDGFRA gene and the DARS2 gene (Figure 3C). Analysing the copy situation, we were surprised to find that 94% of the gene copy number increase is greater than the copy number loss. We could also observe the localization of related genes on the chromosome, through the copy number circle map (Figures 3D, E).

### 3.3 Model construction and verification

By performing lasso regression on 17 prognostically relevant differential genes, we found that 11 genes (ABCC9, AHNAK, CHMP4C, CLIC3, DARS2, DIP2C, PLOD1, POU5F1, RAD9A, RUNX2, and SLC3A2) had minimal error when constructing the model (Supplementary Figures S1, S2). Therefore,

Risk score = EXP [(ABCC9 * 0.104875875974905) + (AHNAK * 0.105361774967532) + (CHMP4C * –0.177580413347782) + (CLIC3 * 0.0643555617499566) + (DARS2 * 0.242694054934441) + (DIP2C * 0.0988937249166662) + (PLOD1 * 0.0520153294064727) + (POU5F1 * -0.0320143641599491) + (RAD9A * –0.234743212057812) + (RUNX2 * 0.0706278491949038) + (SLC3A2 * 0.123190106690304)]

**FIGURE 4:**
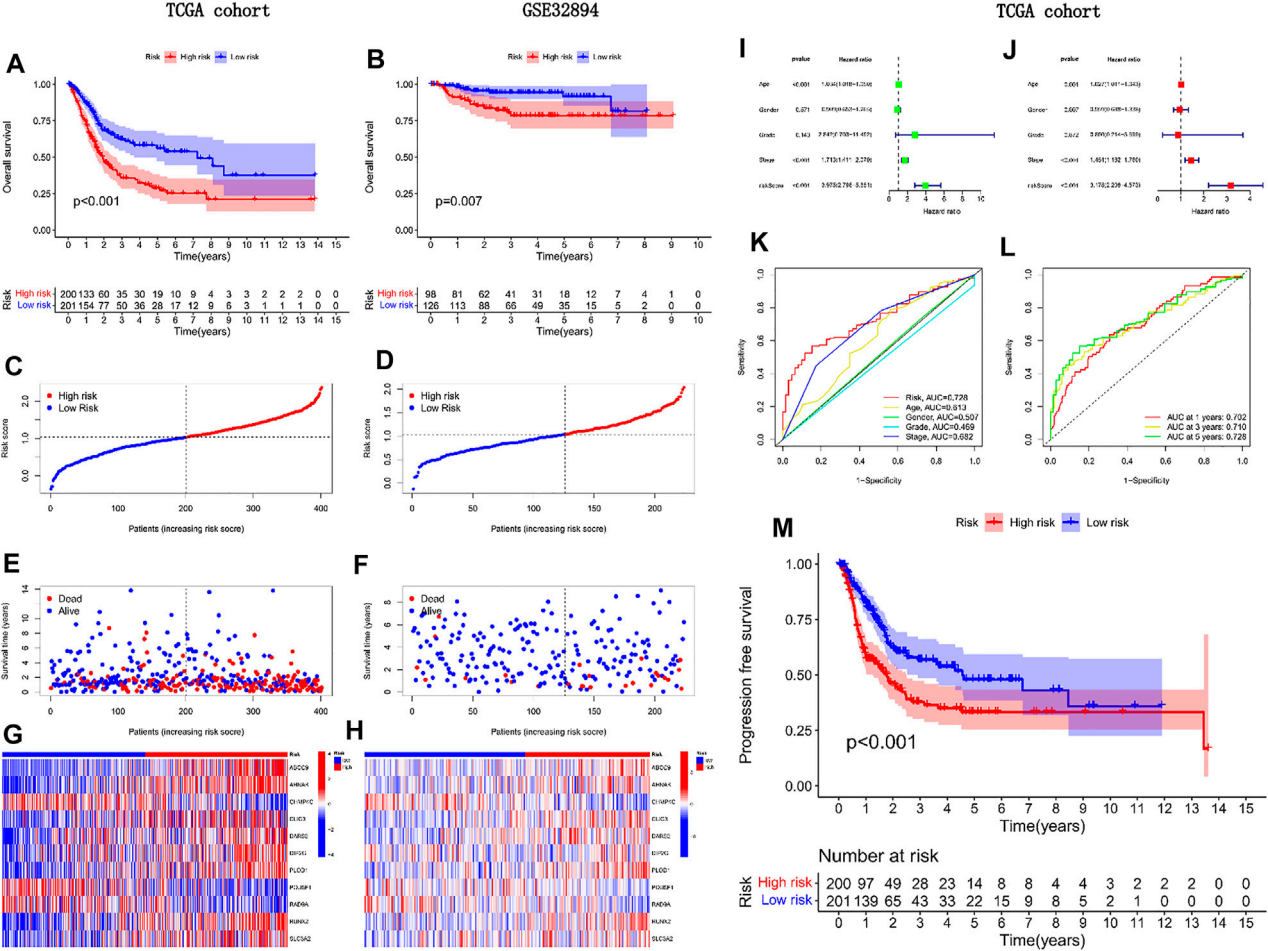
External validation of the predictive power of the model in prognosis.**(A)** Survival analysis of TCGA cohorts; **(B)** Survival analysis of GEO cohorts; **(C, E, G)** Risk curve for TCGA cohorts; **(D, F, H)** Risk curve for GEO cohort; **(I)** Univariate independent prognostic analysis forest plot; **(J)** Multifactorial independent prognostic forest plot; **(K)** Clinical ROC curve; **(L)** Time ROC curve; **(M)** Progression-free survival analysis of TCGA cohorts.

We divided patients in the internal and external groups into A and B groups by scores, and then performed survival analysis. Unless otherwise noted, the median risk score for patients in the internal group was used here to classify all patients into high-risk or low-risk groups. Group A was high risk and Group B was low risk. We found that patients in group A have a worse prognosis in both the internal and external groups (Figures 4A, B). We also observed that, as the risk score increased, so did the number of patient deaths. Mortality was higher in high-risk patients than in low-risk patients (Figures 4C–H). In the forest plot of the single-factor independent prognostic analysis, we observed that the predictive power of the risk score was independent of other factors, and better than other factors (*p* < 0.001, HR = 3.975, Figure 4I). We reached the same conclusion in a multivariate independent prognostic analysis (Figure 4J). The AUC of risk score in the multivariate ROC curve (AUC = 0.728) was much higher than the AUC of other indicators (Figure 4K). In addition, all ROC values obtained in the timieROC curve were above 0.7 (0.702, 0.710, and 0.728, see Figure 4L). Finally, through progression-free survival analysis, we found that patients in group B had longer progression-free survival and better quality of life (Figure 4M). Therefore, a more aggressive treatment plan should be formulated for patients in group B.

### 3.4 The immunohistochemical staining results of model-related genes were consistent with gene expression

**FIGURE 5:**
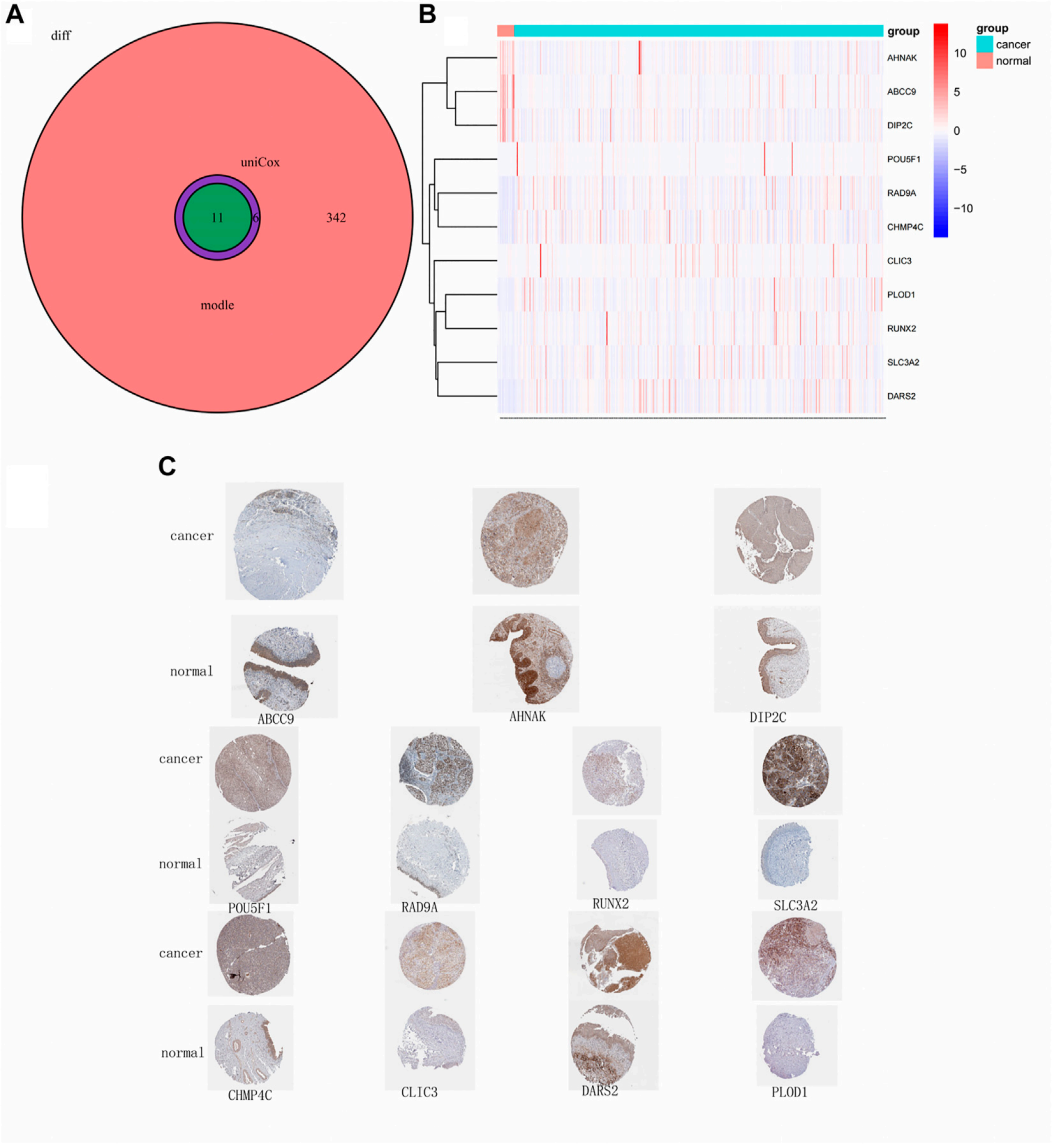
Model-related gene and protein expression.**(A)** Venn plot of model-related genes; **(B)** Heatmap of 11 model-related genes; **(C)** Immunohistochemistry of 11 model-related genes obtained from obtained from the HPA database.

Through rigorous screening, we obtained 11 model-relevant genes (Figure 5A). While three genes (AHNAK, ABCC9, and DIP2C) were highly expressed in normal tissues, eight genes (CHMP4C, CLIC3, DARS2, PLOD1, POU5F1, RAD9A, RUNX2, SLC3A2) were highly expressed in tumour tissues (Figure 5B). By querying the immunohistochemistry of these genes through the HPA database, we found that AHNAK, ABCC9, and DIP2C stained deeper in normal tissues, and CHMP4C, CLIC3, DARS2, PLOD1, POU5F1, RAD9A, RUNX2, SLC3A2 stained deeper in tumour tissues (Figure 5C), which is consistent with their gene expression.

### 3.5 Prognostic models are equally applicable to populations with different clinicopathological features

**FIGURE 6:**
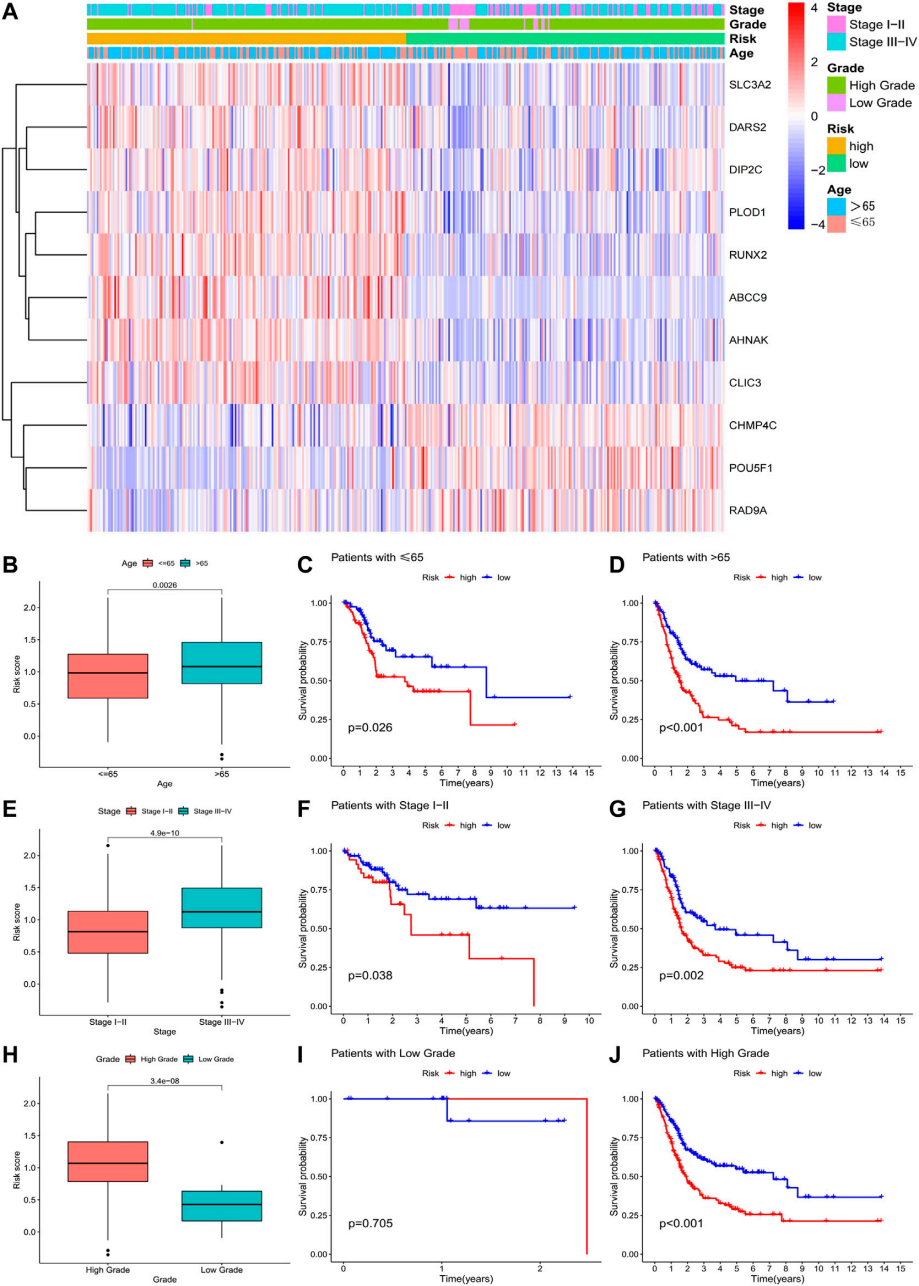
Correlation of risk models with clinical data.**(A)** Clinically relevant heatmap of 11 risk model-related genes; **(B–D)** Boxplot and survival analysis of risk score differences in patients of different ages; **(E–G)** Boxplot and survival in risk scores and survival analysis of patients with different grades. **(H–J)** Boxplot of the difference in risk scores and survival analysis of patients with different grades.

In the clinically relevant heatmap, as well as the boxplot, is noticeable that a patient’s risk increases with age and as the tumour progresses (Figures 6A, B, E, H). Eight genes (SLC3A2, DARS2, DIP2C, PLOD1, RUNX2, ABCC9, AHNAK, and CLIC3) may be involved in the malignant transformation of tumours, and three genes (CHMP4C, POU5F1, and RAD9A) may have a protective effect in patients with tumours (Figure 6A). Survival analysis was performed in patients grouped with different clinicopathological features, and we obtained consistent results, except in patients with low-grade bladder cancer (Figures 6C, D, F, G, J). We examined the data for this phenomenon and found that high-risk patients rarely had low-grade bladder urothelial carcinoma, which was the only case in our data. Conclusions drawn in the absence of sufficient data are justifiably inaccurate. Therefore, the model is also applicable to people with different clinicopathological characteristics.

### 3.6 Drawing of a nomogram

**FIGURE 7:**
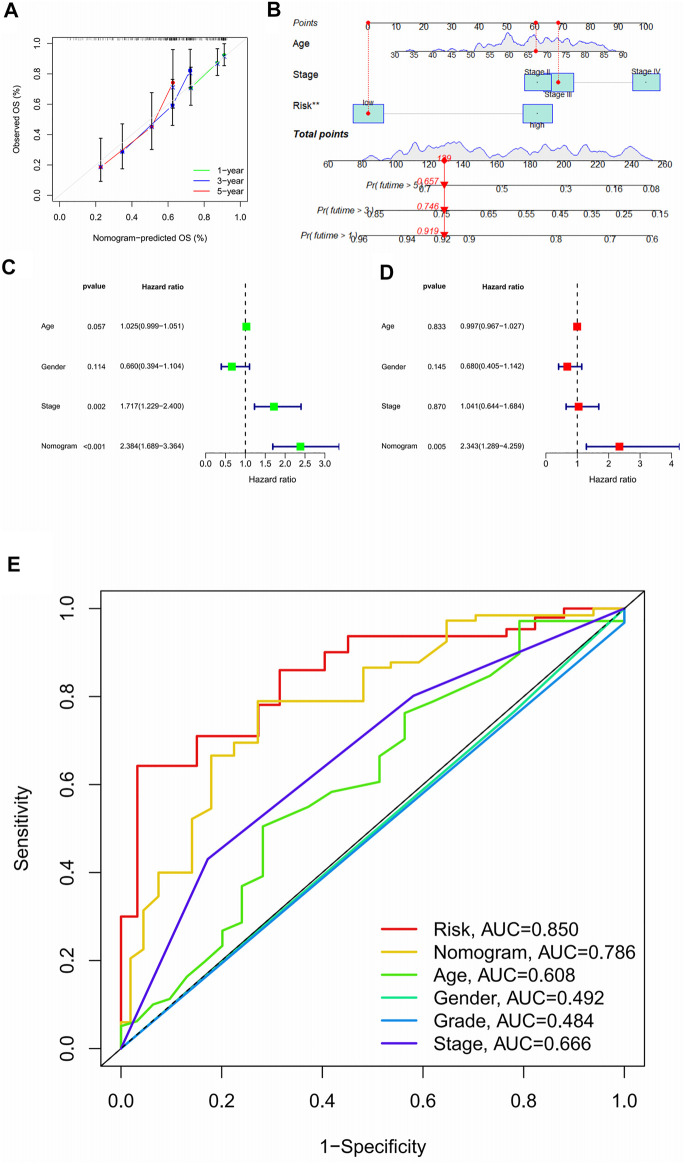
Nomograms are easier for clinical use.**(A)** 1, 3, 5 year calibration curve; **(B)** Nomogram; **(C)** Forest plot for single-factor independent prognostic analysis of nomograms; **(D)** Forest plot for multi-factor independent prognosis analysis of nomograms; **(E)** Nomograms ROC curve.

Through the above research, we have obtained a stable and reliable scoring model. We integrated model scoring with clinically accessible age and tumour stage information, to create a more user-friendly nomogram (Figure 7B). We used the ninth patient in the study cohort as an example to show how to use this nomogram. The survival rate of the patient in the first year is 0.919, in the third year is 0.746, and in the fifth year is 0.657; thereby, the survival rate of the patient decreases year by year, which is consistent with the actual situation in the clinic. For this patient, we should adopt a more active diagnosis and treatment strategy. In the calibration curve, the nomogram calculation for the first year was exactly consistent with the actual survival rate, with the results for the third and fifth years slightly deviating (Figure 7A). In the results of univariate independent prognostic analysis, the nomogram was superior to other clinical indicators (Figure 7C). This conclusion was further confirmed by the results of a multivariate independent analysis (Figure 7D). In the multivariate ROC curve, the result of the nomogram (AUC = 0.786) was second only to the result of the scoring model (AUC = 0.850, see Figure 7E).

### 3.7 Pathway enrichment of GSVA, GO, and KEGG

**FIGURE 8:**
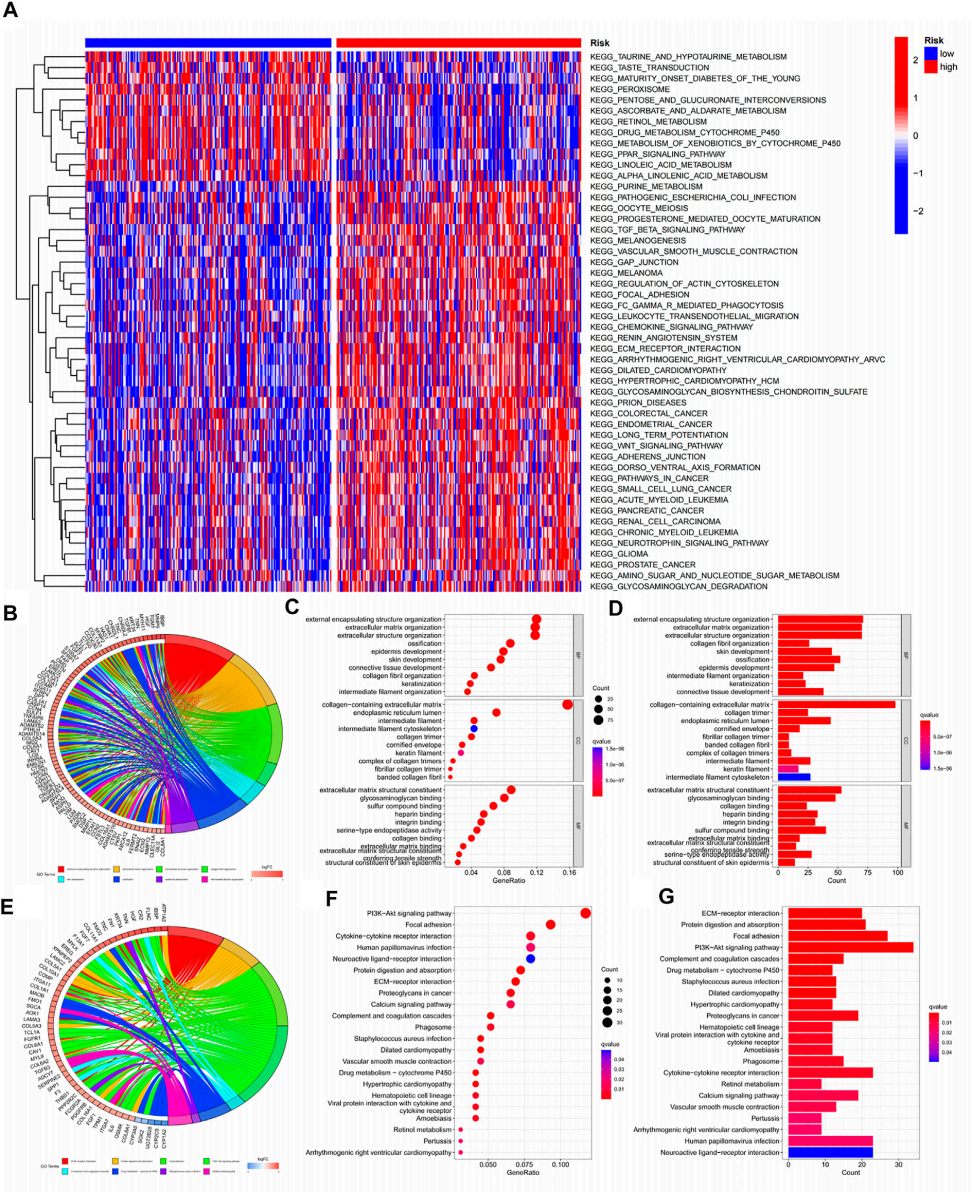
Pathway enrichment of GSVA, KEGG, GO.**(A)** Pathway enrichment heat map for GSVA analysis; **(B–D)** Circle chart, significance bubble chart, significance histogram for GO analysis; **(E–G)** Circle plot, significance bubble chart, significance histogram for KECG analysis.

As tumours progress, their metabolism and function become more complex. In previous analyses, we found that model scores were positively correlated with tumour progression. This conclusion was confirmed by the GSVA analysis heatmap, where 24% of pathways were active in regions with low model scores, and 76% were active in regions with high model scores (Figure 8A). Most of the active pathways in the regions with low model scores involve tissue cell metabolism, while the active pathways in regions with higher model scores are closely related to a variety of tumorigenesis and immune drives. Thus, the changes in the early stage of tumours occur mainly at the metabolic level, and various tumours may undergo similar processes in the terminal stage. It is then feasible to speculate that changes in metabolic levels may be the key to the early diagnosis of tumours. For advanced tumours, other types of treatment solutions may have reference implications. Most of the pathways obtained by GO analysis were related to extracellular matrix components and structures (Figures 8B–D). A total of 22 pathways were enriched by KEGG analysis (Figures 8E–G).

### 3.8 Suitable treatment modalities for advanced tumours are more diverse

**FIGURE 9:**
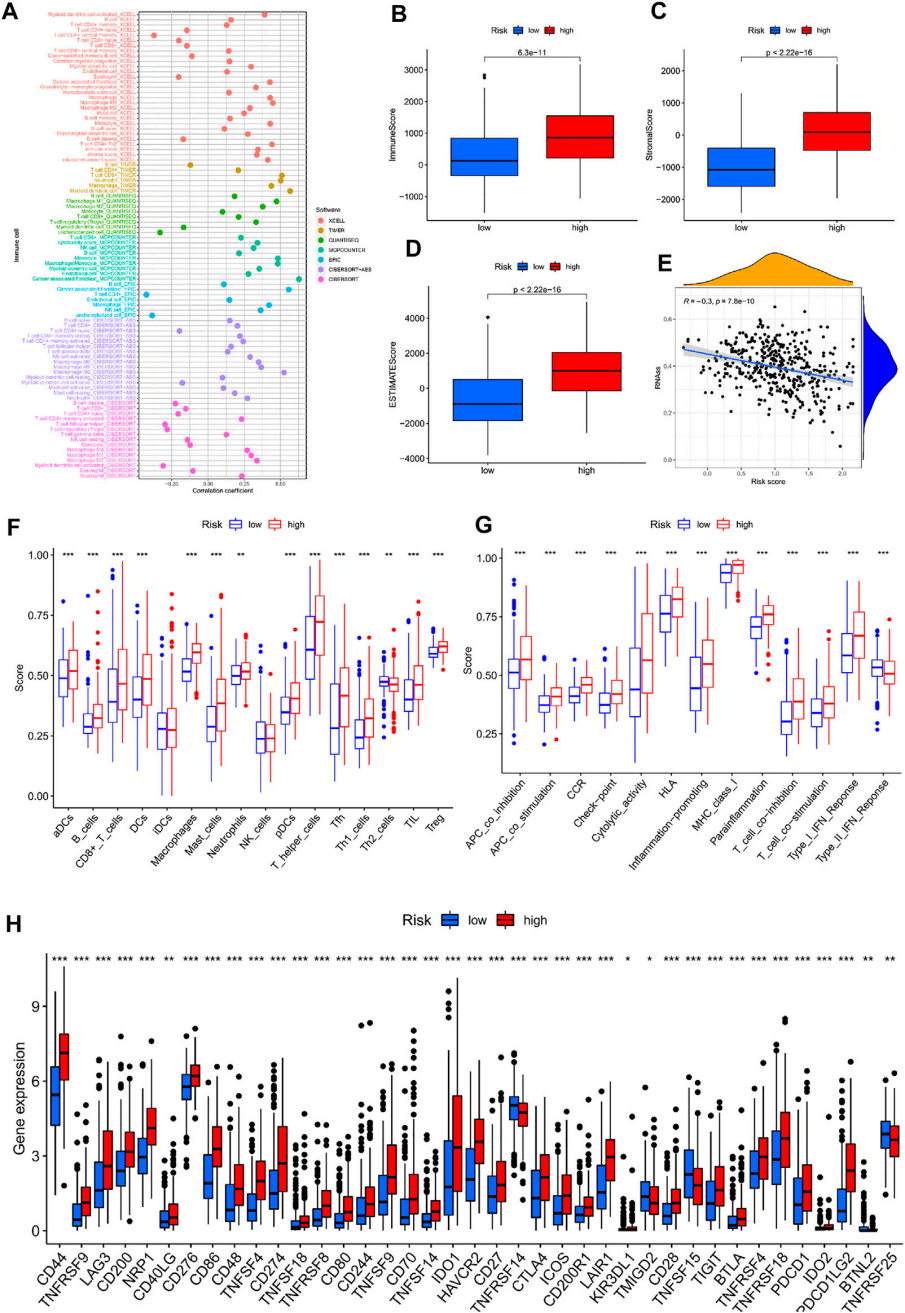
Immunocorrelated analysis between high-and low-risk groups; **(A)** Immune cell correlation analysis bubble map; **(B–D)** Box plot of tumor microenvironment analysis; **(E)** Scatterplot of stem cell correlation analysis; **(F)** ssGSEA analysis of immune cell infiltration box plot; **(G)** ssGSEA analysis of immuno-related function enrichment boxplot; **(H)** Box plot of gene expression difference associated with immune checkpoints in high-and low-risk groups.

In previous pathway enrichment analysis, we found that advanced tumour immune pathways were more abundant, and we surmised that this would help in selecting advanced tumour treatment modalities. This hypothesis was further validated in subsequent analyses. From the analysis of seven platforms, we estimated that 67% of immune cells had a correlation coefficient greater than 0 with the model score (Figure 9A). A further analysis of the tumour microenvironment yielded the same conclusion (Figures 9B–D). In stem cell correlation analysis, we found that bladder cancer had strong stem cell characteristics in the early stage, which may be related to the recurrent nature of bladder cancer (Figure 9E). To understand which cells had increased infiltration in advanced tumour tissue, we presented the results as boxplots by ssGSEA analysis (Figure 9F). As expected, 87.5% of immune cells were significantly elevated in advanced tumours. Correspondingly, all immune-related functions were active in advanced tumours (Figure 9G). To explore the causes for this, we analysed the immune checkpoint gene (Figure 9H). Incredibly, 74% of the immune checkpoint genes we analysed were highly expressed in advanced tumours. So, immune checkpoint inhibitors may be a good option in patients with advanced bladder cancer. Other immunotherapy modalities may also be valuable in patients with advanced bladder cancer. The sensitivity analysis for chemotherapy drugs in patients with bladder cancer showed that patients with advanced bladder cancer had a wider choice of chemotherapy drugs (Supplementary Figure S3). When tumours enter the advanced stage, it is often difficult to achieve good results with a single treatment method. Through our model, we have explored a variety of treatment options that have significant implications for patients with advanced tumours.

### 3.9 Identification of subtypes to further explore treatment options

Referring to the matrix heatmap, delta area plot, consistency cumulative distribution function plot, and tracking plot, we divided the sample into two subtypes (Supplementary Figures S4A–D). From the Sankey plot, we observed that patients with the C1 subtype had only minor differences when compared with patients with advanced tumours, and patients with the C2 subtype intersect with those with advanced tumours (Supplementary Figure S4E). The PCA diagram for both was well distinguished (Supplementary Figures S4F, G). The clinically relevant heatmap shows the distribution of different clinical features across subtypes (Supplementary Figure S4H). Although the distribution of patients in the subtype differed slightly from those in the model, the survival of patients with the C2 subtype was still higher than that of the C1 subtype (Supplementary Figure S4I).

We further explored treatment options for subtypes using similar analytical methods. When exploring immunotherapy options, we obtained the same results as before, except that the infiltration of individual immune cells differed (Supplementary Figure S5). As further analysis of the specific mechanisms of the results might reveal the cause for this, we performed drug susceptibility analysis, and obtained fewer sensitive drugs than before. This demonstrates that the subtyping of bladder cancer has limited effect for the precise use of chemotherapy drugs (Supplementary Figure S6).

## 4 Discussion

Telomere maintenance mechanisms are widespread in bladder cancer. In this study, we used a public database to establish a scoring model for telomere maintenance genes. After external data verification and querying the immunohistochemistry of model genes, the results obtained by the model were considered stable and reliable. By grouping patients with different clinicopathological features, we further confirmed that the model is equally reliable in different populations. In addition, we plotted nomograms, to make the model easier to use in the clinic. Just by scoring this model and some information that is commonly available in the clinic, we can predict the multi-year survival rate of each patient. The treatment plan is often different for patients with different survival periods, so our research may be helpful for individualized treatment. For patients who are expected to survive longer, we often need to adopt a more positive attitude. For patients with a small expected survival period, palliative care is most often required, to relieve patient suffering and save medical resources. After obtaining the expected survival of patients, combined with the results of the analysis of immune characteristics and the sensitivity analysis of chemotherapy drugs, formulating appropriate treatment methods for patients at different stages of tumour development would be possible. The differentiation between different subtypes of tumours was conducted to further explore the options of precision treatment. Unfortunately, this study did not have the capacity to conduct in-depth research on specific immune mechanisms, as the data for the study was generated from public databases. Although this study was thorough in the verification of the predictive ability of nomograms, it still requires practical clinical research as theoretical support.

The pathways related to metabolism were mainly enriched in the regions with low model scores, while the pathways related to immunity and tumours were mainly enriched in regions with high model scores. This suggests that the characteristics of tumour tissue are gradually changing as the model score increases (i.e., tumour progression). This indicates that early tumours may only change in metabolism, and through this phenomenon we can look for some characteristic metabolites for early diagnosis of tumours. On the other hand, advanced tumours have similar development to other tumours, and local invasion of immune cells is more pronounced. A single treatment for advanced tumours often has little effect, and this feature allows us to draw inspiration from effective treatment options in other cancers, to enrich the treatment of advanced bladder cancer. Immunotherapy and multi-agent chemotherapy regimens are expected to enrich the diagnosis and treatment of patients with advanced bladder cancer.

Through the HPA database, we found that three genes (ABCC9, AHNAK, and DIP2C) had low expression in patients with tumours, whereas eight genes (PLOD1, SLC3A2, RUNX2, RAD9A, CHMP4C, DARS2, CLIC3, and POU5F1) were highly expressed.

In a tissue microarray study of 87 primary urothelial carcinoma and 17 control cases, we found that RUNX2 independently predicted early tumour recurrence in patients with bladder urothelial carcinoma (Abdelzaher and Kotb, 2016). An *in vivo* study also found that microRNA-154 could inhibit cellular malignancies by targeting RUNX2 (Zhao et al., 2017). Other studies found that CHMP4C can not only promote the malignant development of cervical cancer cells, but also regulate the occurrence and progression of lung squamous cell carcinoma through the cell cycle pathway (Lin et al., 2020; Liu et al., 2021). CHMP4C has also been shown to be a prognostic marker for cervical cancer (Hu et al., 2022). Based on the correlation between tumour cells, we can speculate that CHMP4C is equally reliable as a prognostic marker for bladder cancer. DARS2 is not only a gene related to telomere maintenance, but also an RBP gene (Gerstberger et al., 2014). As such, DARS2 can be used as a prognostic marker for bladder cancer (Guo et al., 2020; Wu et al., 2021). CLIC3 is highly expressed in bladder cancer and is a marker of poor prognosis in patients with bladder cancer (Chen et al., 2020). Previous studies have shown that POU5F1 is highly expressed in bladder cancer, which is consistent with our findings (Atlasi et al., 2007). POU5F1 is overexpressed in tumour cells and may be associated with tumour progression and metastasis (Chang et al., 2008). POU5F1 can enhance tumour immune response by upregulating the TET1-dependent NRF2/MDM2 axis in bladder cancer (Mao et al., 2021).

## 5 Conclusion

In this study, we constructed a reliable prognostic model that can accurately predict patient prognosis. Our search for precision therapy has the potential to enrich the treatment options for patients with advanced bladder cancer.
