# Supplementary material for: Telomere maintenance-related genes are important for survival prediction and subtype identification in bladder cancer
Source: Front Genet. 2023 Jan 6;13:1087246. doi: 10.3389/fgene.2022.1087246 (PMC9853053; doi:10.3389/fgene.2022.1087246)
Supplement: Supplementary file 1 [file Table1.DOCX]

Supplementary Material

# Supplementary Figures


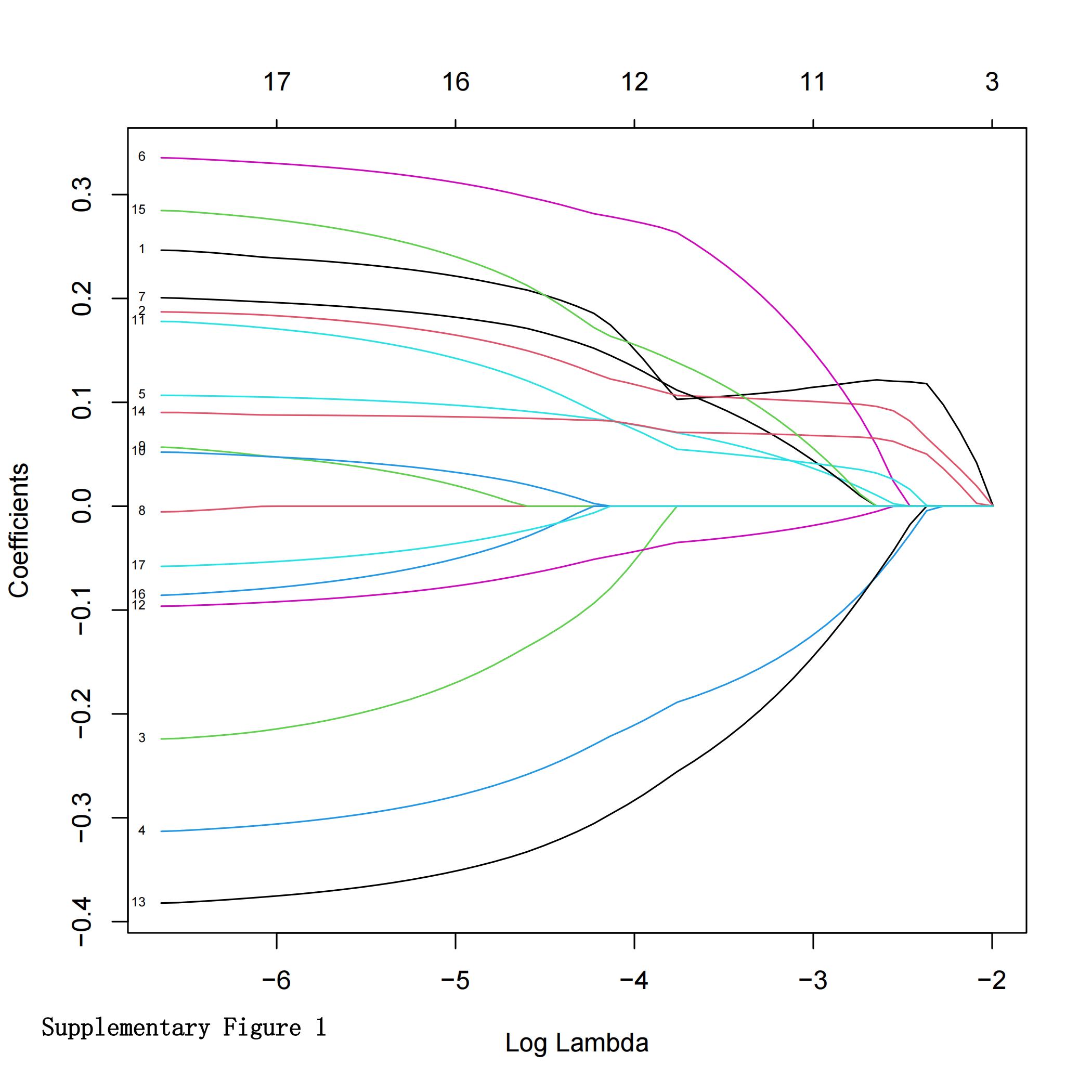


**Supplementary Figure 1.** Log Lambda.


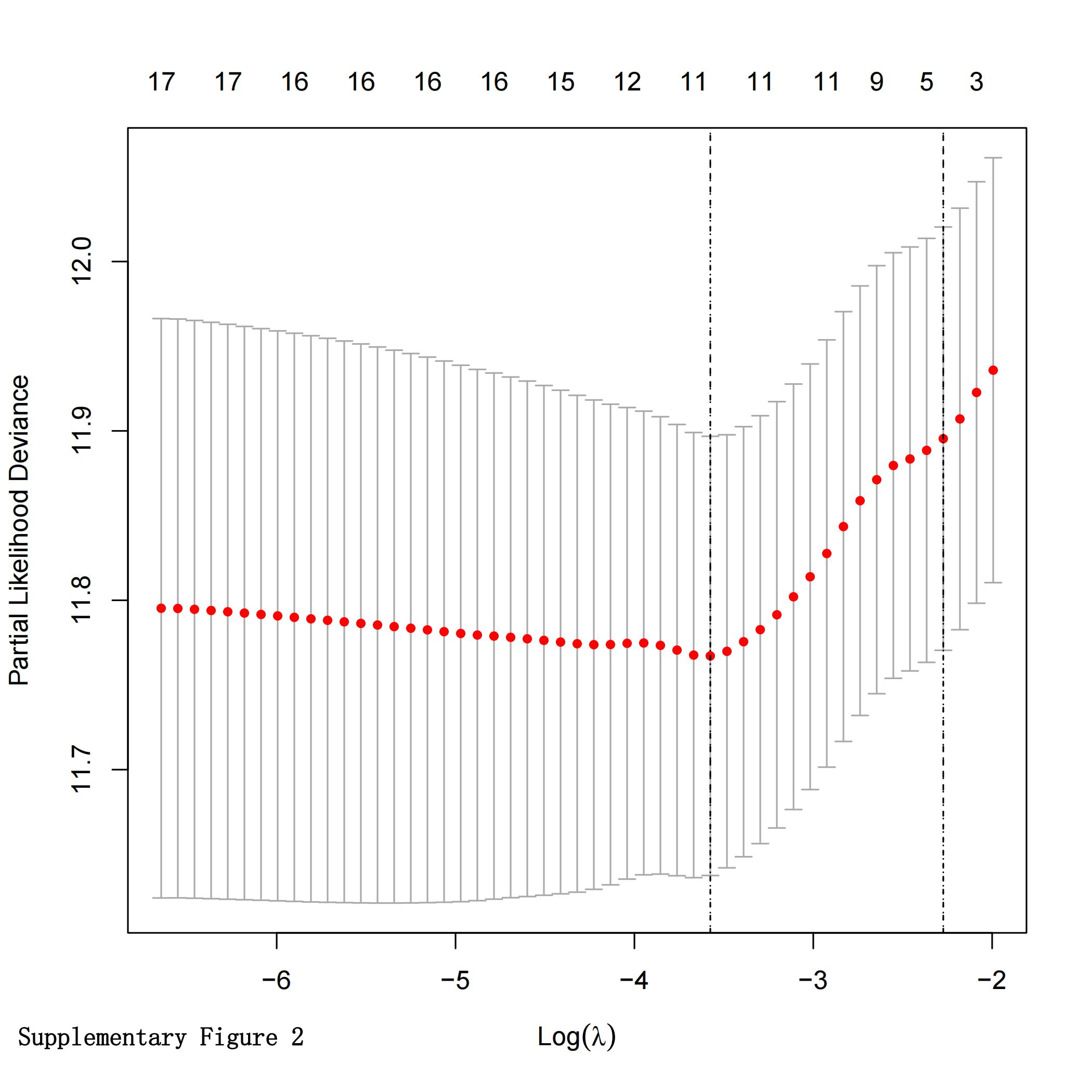


**Supplementary Figure 2.** Select the location with the least error.


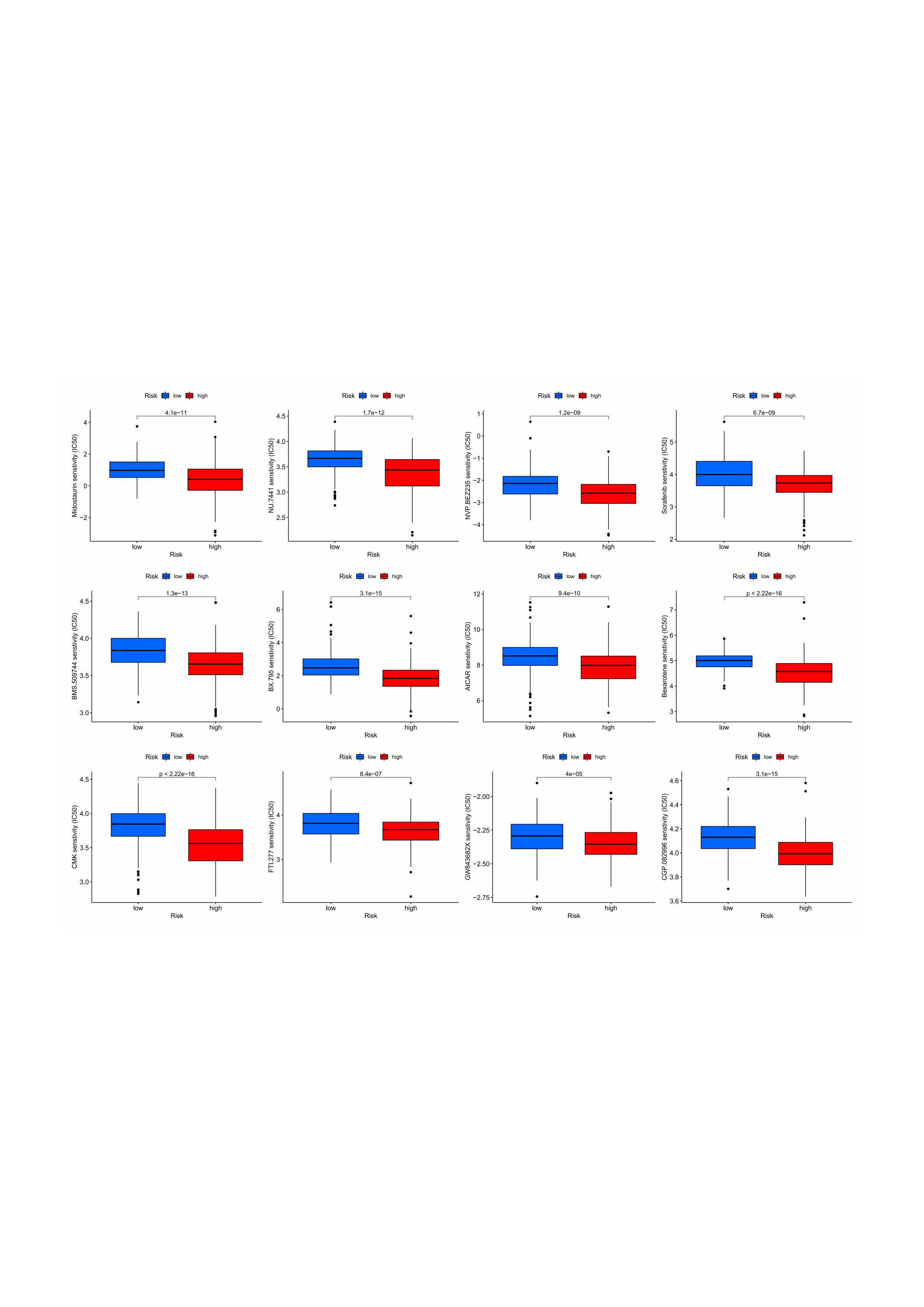


**Supplementary Figure 3.** Partially sensitive chemotherapy drugs for advanced tumors.


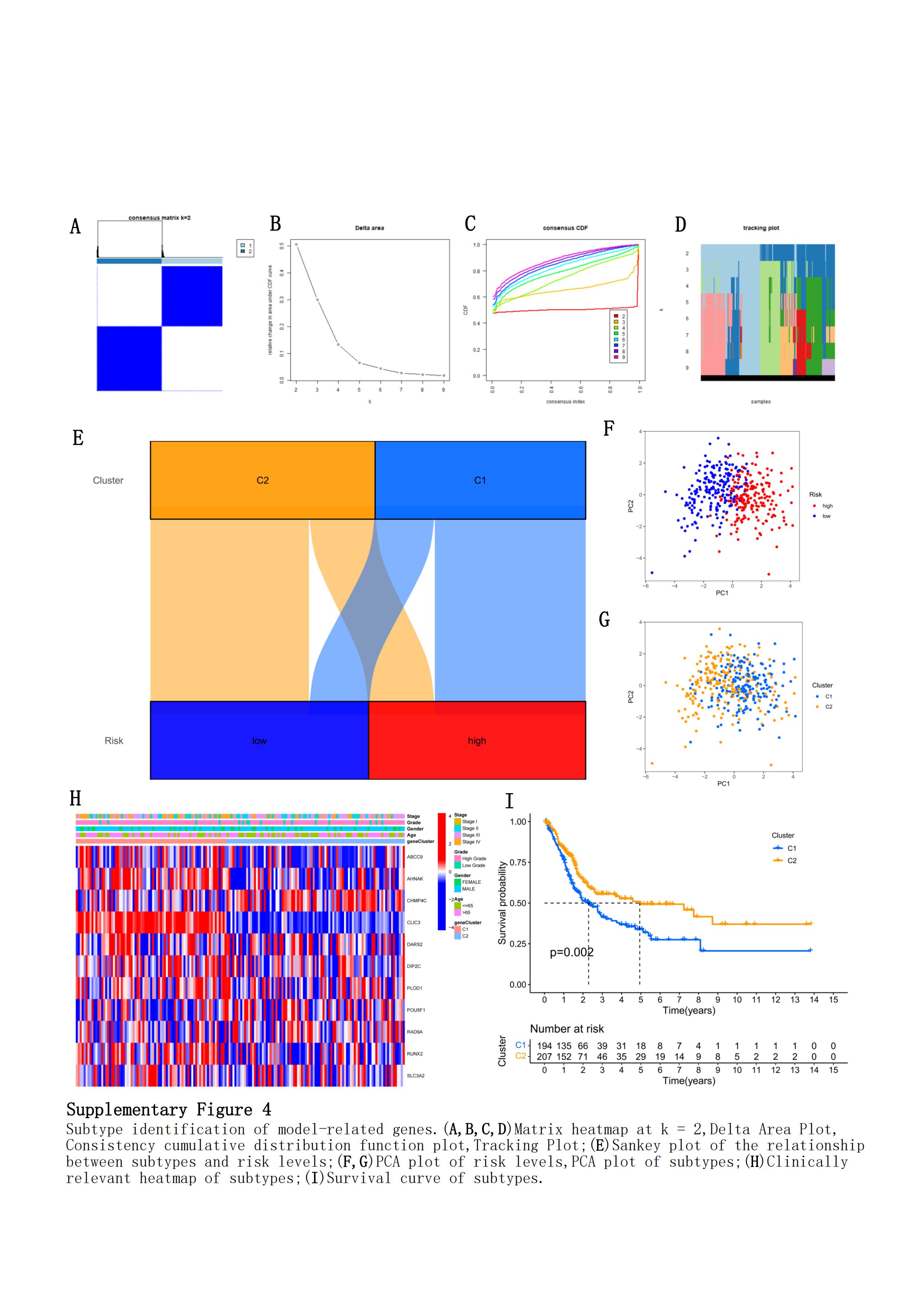


**Supplementary Figure 4.** Subtype identification of model-related genes.(**A,B,C,D**)Matrix heatmap at k = 2,Delta Area Plot, Consistency cumulative distribution function plot,Tracking Plot;(**E**)Sankey plot of the relationship between subtypes and risk levels;(**F,G**)PCA plot of risk levels,PCA plot of subtypes;(**H**)Clinically relevant heatmap of subtypes;(**I**)Survival curve of subtypes.


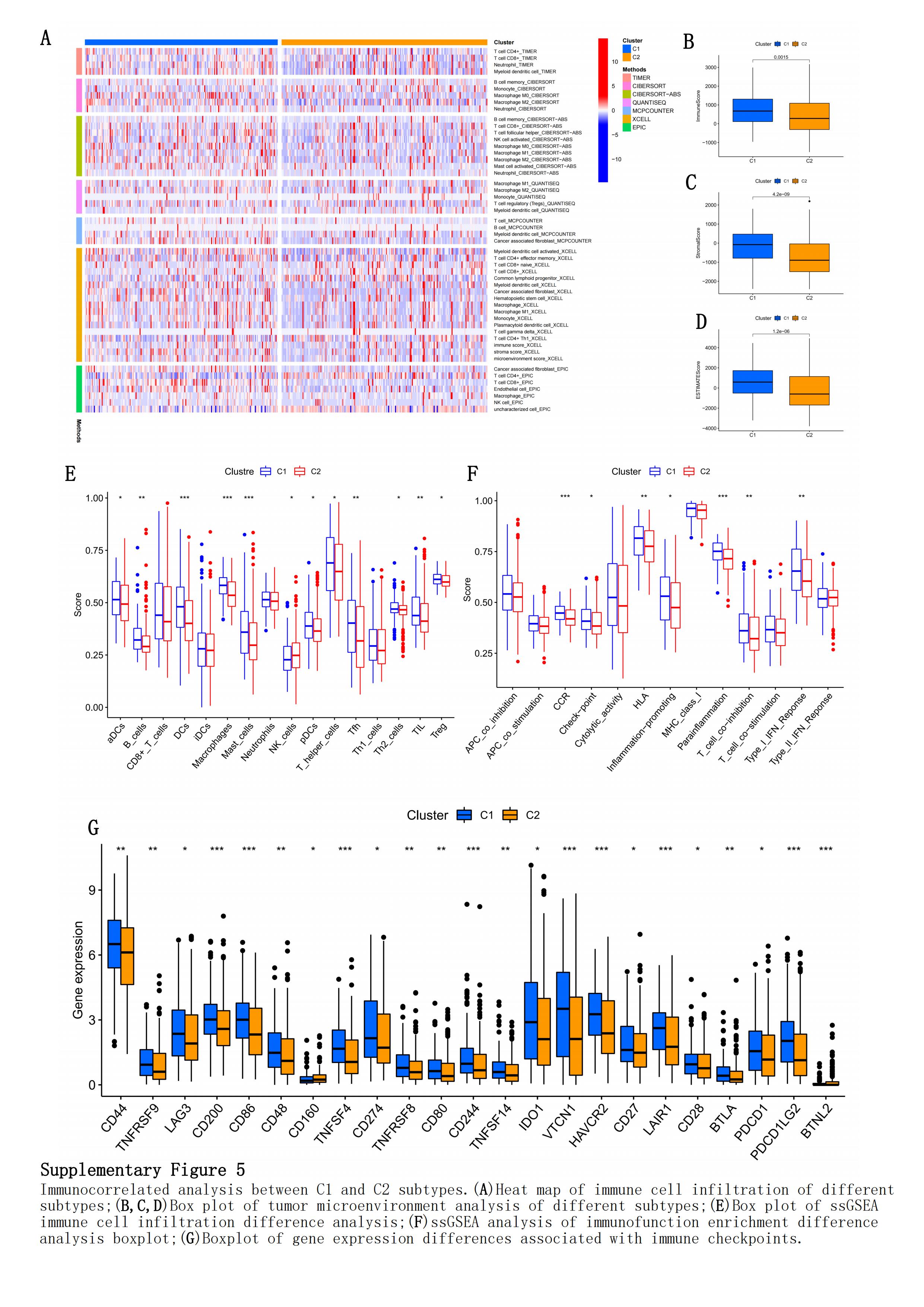


**Supplementary Figure 5.** Immunocorrelated analysis between C1 and C2 subtypes.(**A**)Heat map of immune cell infiltration of different subtypes;(**B,C,D**)Box plot of tumor microenvironment analysis of different subtypes;(**E**)Box plot of ssGSEA immune cell infiltration difference analysis;(**F**)ssGSEA analysis of immunofunction enrichment difference analysis boxplot;(**G**)Boxplot of gene expression differences associated with immune checkpoints.


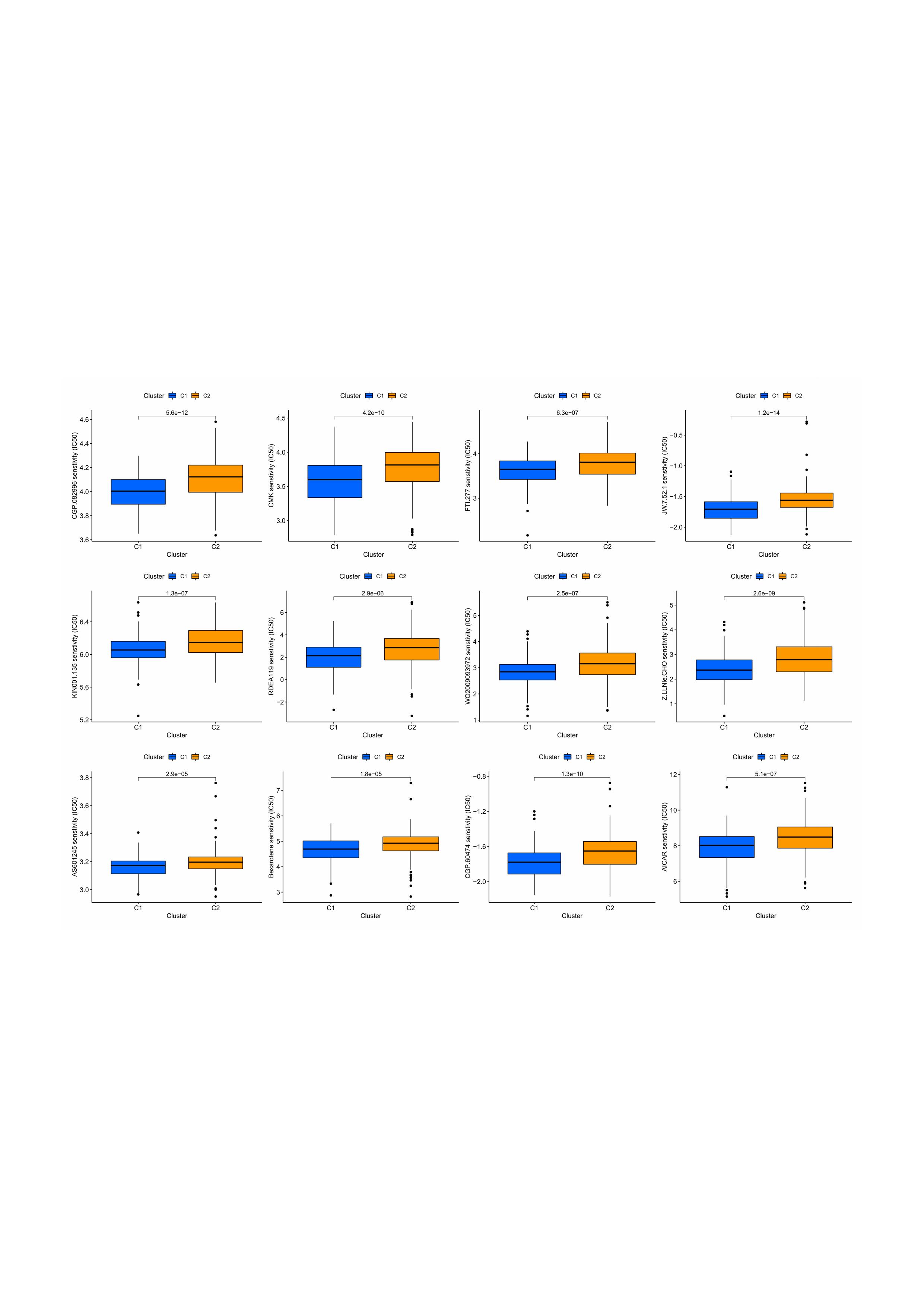


**Supplementary Figure 6.** C1 subtype partially sensitive chemotherapy drugs.
